# The complete mitochondrial genome of *Cerithidea sinensis* (Philippi, 1848)

**DOI:** 10.1080/23802359.2019.1644549

**Published:** 2019-07-24

**Authors:** Youhou Xu, Peng Luo, Pengliang Wang, Peng Zhu, Hong Zhang, Haiping Wu, Yongyan Liao, Minhang Yu, Jirui Fu

**Affiliations:** a Guangxi Key Laboratory of Beibu Gulf Marine Biodiversity Conservation, Beibu Gulf University, Qinzhou, Guangxi Autonomous Regions, China;; b Key Laboratory of Tropical Marine Bio-resources and Ecology (LMB), South China Sea Institute of Oceanology, Chinese Academy of Sciences, Guangzhou, Guangdong, China;; c College of Animal Science and Technology, Guangxi University, Nanning, Guangxi, China

**Keywords:** *Cerithidea sinensis*, mitochondrial genome, Illumina sequencing

## Abstract

*Cerithidea sinensis* is a common and important component of mangrove ecosystem. In this study, the mitochondrial genome of *C. sinensis* was determined for the first time using next-generation sequencing; the overall base components of mitogenome consisting of 15633 bp was 31.14% for A, 35.70% for T, 16.65% for G, 16.51% for C, and its GC content was 33.16%. The mitochondrial circular genome was composed of 13 protein-coding genes, 22 tranfer RNAs, and 2 ribosomal RNAs. Polygenetic analysis showed that the *C. sinensis* was more closed to *Semisulcospira libertina* than *Turritella bacillum* and *Tylomelania sarasinorum*. We may speculate that the *C. sinensis* is evolved from freshwater species.

The *Cerithidea sinensis* is a member of the genus *Cerithidea*, which is common and important component of mangrove forests, tidal swaps, and salt marshes in the coast of south china sea, in marine and brackish conditions (Reid 2014). Figuring out how the *C. sinensis* co-evolved in the mangrove ecosystem requires a deeper understanding of molecular genetics. The genetic makers from mitochondrial DNA were very effective, which could successfully be applied to the study of population genetics, molecular phylogenetics, and evolutionary studies. Hence, the complete mitochondrial genome sequence of *C. sinensis* was sequenced, assembled, and characterized, which could provide important genetic data for elucidating the evolution relationship of genus *Cerithidea*.

Total genomic DNA was isolated from each species using approximately the muscle tissue. Total DNA was eluted in sterile deionized water and was stored at −20 °C. The specimen was collected from Shajing Gang region, Qinzhou City, province Guangxi, China (21.838N, 108.602E) and stored at Herbarium of Ocean College in Beibu Gulf University (C.S.002). Paired-end library (450 bp) was sequenced using Illumina Hiseq4000 platform, with 150 bp pair-end sequencing method. The mitochondrial genome assembly using the chloroplast and mitochondrion assemble (CMA) V1.1.1 software (Guangzhou SCGene Co., Ltd, Guangzhou, Guangdong, China, http://www.scgene.com), which was based on sequencing reads’ overlap and paired-end relationship. Protein-coding genes and rRNA genes were annotated with blast+(2.5.0) with allied species, and tRNAs were predicted with tRNAscan-SE v2.0 (http://lowelab.ucsc.edu/tRNAscan-SE/) (Lowe and Chan 2016). 7700 raw reads with average length of 150 bases and 1,155,000 nt was obtained with average reads depth of 73.8X. Our research findings revealed that the circular genome is 15,633 bp, which consists of 13 protein-coding genes(PCGs), 2 rRNAs genes, 22 tRNAs genes, showing that the gene composition and arrangement are more close to reported *S. libertina* (Zeng et al. 2015). The contents of A, T, G, and C in mitochondrial genome were 31.14, 35.70, 16.65, 16.51%, respectively. An overall GC content of whole mitochondrial genome is 33.16%. The sequence was deposited in GenBank (GenBank: KY021067). A phylogenetic analysis was conducted on 15 mitochondrial genomes from Prosobranchia and an entire mitogenome of *Pomacea canaliculata* as an outgroup. Maximum likelihood (ML) method was used for phylogenetic analysis. The best-fit models of evolution for the coding genes were selected by jmodeltest2 (https://github.com/ddarriba/jmodeltest2) (Darriba et al. 2012). RAxML v8.0.0 (Stamatakis 2014) was used to build the tree with 1000 bootstrap (Figure 1). Phylogenetic analyses indicated the presence of two distinct clades in Prosobranchia and showed that the *C. sinensis* was more close to *S. libertina* than *T. bacillum* and *T. sarasinorum*, the former lives in freshwater and the two later in brackish water. We may speculate that the *C. sinensis* is evolved from freshwater species. The compete mitochondrial genome of *C. sinensis* provide important genetic information for understanding phylogenetic relationships of Prosobranchia mitochondrial genome and will be useful for figuring out how the *C. sinensis* co-evolved in the mangrove ecosystem.

**Figure 1.**
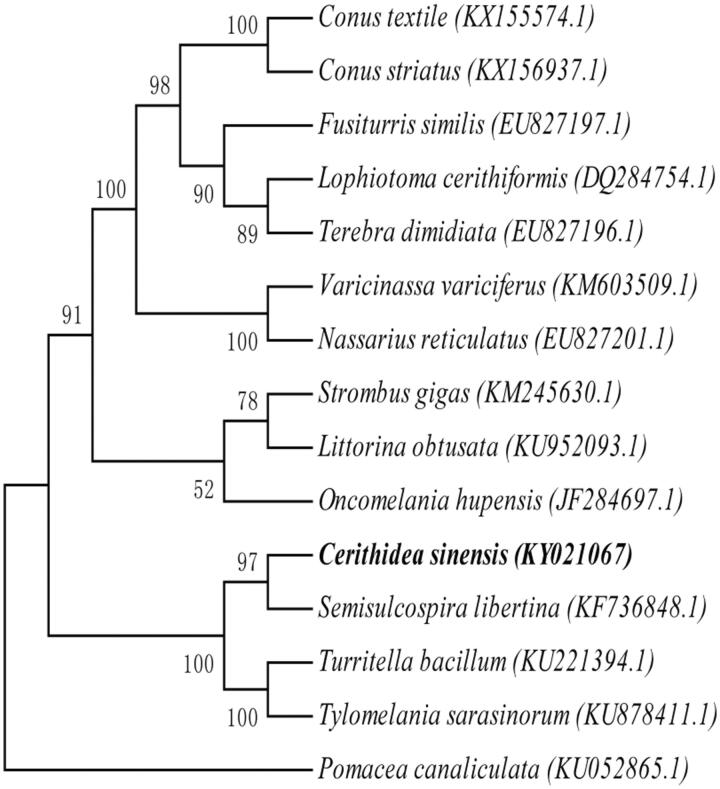
Phylogenetic relationships of Prosobranchia based on 15 mitochondrial genomes using NJ method. GenBank accession numbers: *Conus textile* (KX155574.1), *Conus striatus* (KX156937.1), *Fusiturris similis* (EU827197.1), *Lophiotoma cerithiformis* (DQ284754.1), *Terebra dimidiata* (EU827196.1), *Varicinassa variciferus* (KM603509.1), *Nassarius reticulatus* (EU827201.1), *Strombus gigas* (KM245630.1), *Littorina obtusata* (KU952093.1), *Oncomelania hupensis hupensis* (JF284697.1), *Semisulcospira libertina* (KF736848.1), *Turritella bacillum* (KU221394.1), *Tylomelania sarasinorum* (KU878411.1), *Pomacea canaliculata* (KU05286.1), *Cerithidea sinensis* (KY021067).
